# Correspondence on “Direct potable reuse and birth defects prevalence in Texas”: An augmented synthetic control method analysis of data from a population-based birth defects registry

**DOI:** 10.1097/EE9.0000000000000346

**Published:** 2024-10-10

**Authors:** Daniel Gerrity, Joseph N.S. Eisenberg, Charles P. Gerba, Keisuke Ikehata, Kristina Mena

**Affiliations:** a Applied Research and Development Center, Southern Nevada Water Authority, Las Vegas, Nevada; b School of Public Health, University of Michigan, Ann Arbor, Michigan; c Department of Soil, Water, and Environmental Science, The University of Arizona, Tucson, Arizona; d Ingram School of Engineering, Texas State University, San Marcos, Texas; e School of Public Health, UTHealth Houston, El Paso, Texas

Schraw et al^1^ evaluated birth defect prevalence upon implementation of direct potable reuse (DPR) in Texas. With this correspondence, we would like to emphasize the major finding of this study—that there was no significant difference in birth defect prevalence between DPR and non-DPR counties—and also highlight the safety and reliability of DPR. Broader implementation of DPR is expected throughout the United States based on Texas’ success, as supported by an abundance of water quality and operational data,^2,3^ and extensive research and regulatory development in other states.^4^ We are concerned that the authors’ references to “increases in birth defect prevalence” may be misinterpreted, potentially compromising future efforts to implement potable reuse. This compels us to consider the plausibility of risk mechanisms for Texas’ DPR systems.

DPR involves the use of advanced treatment to purify recycled water before drinking water supply augmentation. This level of advanced treatment often improves drinking water quality compared to conventional source waters.^2,5^ In 2013, the Raw Water Production Facility operated by the Colorado River Municipal Water District (CRMWD) in Big Spring, Texas, became the first facility in the United States to implement DPR. On average, the Raw Water Production Facility produces 5700 m^3^/day, which comprises less than 3% of CRMWD’s overall distributed supply.^6^ In 2014, Wichita Falls, Texas, rapidly implemented its own DPR system in response to severe drought conditions, but because of record rainfall in 2015, this system was operated in a DPR configuration for only 12 months.^3^ In contrast with CRMWD, the Wichita Falls system was designed to provide 50% of the water supply for nearly 100% of the service area population. However, because of its short-term operation, Schraw et al^1^ designated Wichita County as “unexposed to DPR” for their statistical evaluation. Importantly, the authors did not confirm whether pregnant women in any Texas counties used tap water or in what capacity, and the authors’ methods forced them to focus on counties with more limited DPR influence.

Schraw et al^1^ highlighted several specific contaminants, including arsenic and nitrate, as being linked to birth defects but did not indicate whether they were detected in DPR product water, or how concentrations compared to more conventional drinking water supplies. Arsenic and nitrate are known to occur frequently and at high concentrations in west Texas groundwater,^7,8^ and this can lead to elevated exposures for communities that are dependent on these groundwater supplies, particularly private wells not under the purview of a public water system.^7^ Public water systems in the United States (e.g., CRMWD) must ensure that their finished drinking water, regardless of source, complies with the US Environmental Protection Agency’s primary maximum contaminant levels, such as those established for arsenic and nitrate. Moreover, the advanced treatment trains associated with DPR are capable of addressing a wide range of regulated and unregulated contaminants.^2^ CRMWD’s system employs a multi-barrier advanced treatment train consisting of microfiltration, reverse osmosis, and an ultraviolet advanced oxidation process. The purified water is then blended with raw surface water (instantaneous maximum 50/50 blend) before conveyance, additional blending, and conventional drinking water treatment. Based on published studies^9^ and extensive water quality testing at Big Spring^2^ and Wichita Falls,^3^ this treatment approach has been shown to achieve low concentrations of arsenic, nitrate, and various constituents of emerging concern. In fact, the DPR train in Big Spring often produces a higher quality product water than the surface water with which it is blended.^2^ Schraw et al^1^ state that DPR’s “lack of an environmental buffer could conceivably result in increased concentrations of contaminants that have been linked to birth defects and adverse pregnancy outcomes,” but the available evidence suggests that DPR actually protects against these potential exposures.

It is the primary mission of industry professionals to ensure that drinking water systems are protective of public health, so we share the belief from Schraw et al^1^ that ongoing research is warranted to improve our understanding and ability to provide high-quality drinking water. Our goal with this correspondence is to highlight that the past implementation of DPR in Texas—and soon other locations throughout the United States—is the culmination of decades of research and regulatory development. We appreciate the authors interest in studying this issue and for Environmental Epidemiology valuing studies that report “persuasively null results”^10^ in an effort to minimize bias in academic research. We believe the study developed by Schraw et al^1^ is another step in demonstrating that DPR is a viable option for water-stressed communities to achieve a safe and reliable water supply.

## Conflicts of interest statement

The authors declare that they have no conflicts of interest with regard to the content of this report.
